# Correlation of Magnetomechanical Coupling and Damping in Fe_80_Si_9_B_11_ Metallic Glass Ribbons

**DOI:** 10.3390/ma16144990

**Published:** 2023-07-14

**Authors:** Xu Zhang, Yu Sun, Bin Yan, Xin Zhuang

**Affiliations:** 1 Aerospace Information Research Institute, Chinese Academy of Sciences, Beijing 100190, China; 2 Key Laboratory of Electromagnetic Radiation and Sensing Technology, Chinese Academy of Sciences, Beijing 100190, China; 3 School of Physical Science and Technology, Inner Mongolia University, Hohhot 010021, China; 4 University of Chinese Academy of Sciences, Beijing 100049, China

**Keywords:** Fe-based amorphous alloys, magnetomechanical coupling, damping factor

## Abstract

Understanding the correlation between magnetomechanical coupling factors (*k*) and damping factors (*Q^−^*^1^) is a key pathway toward enhancing the magnetomechanical power conversion efficiency in laminated magnetoelectric (ME) composites by manipulating the magnetic and mechanical properties of Fe-based amorphous metals through engineering. The *k* and *Q^−^*^1^ factors of FeSiB amorphous ribbons annealed in air at different temperatures are investigated. It is found that *k* and *Q^−^*^1^ factors are affected by both magnetic and elastic properties. The magnetic and elastic properties are characterized in terms of the magnetomechanical power efficiency for low-temperature annealing. The *k* and *Q^−^*^1^ of FeSiB-based epoxied laminates with different stacking numbers show that a −3 dB bandwidth and Young’s modulus are expressed in terms of the magnetomechanical power efficiency for high lamination stacking.

## 1. Introduction

In recent years, Fe-based amorphous alloys with significant magnetomechanical effects have been successfully used in laminated magnetoelectric (ME) composites, which usually consist of piezoelectric and magnetostrictive layers [1,2,3,4,5]. The ME energy coupling of laminated piezoelectric/magnetostrictive composites can be described as magnetic energy converted to mechanical energy and then to electric energy through the strain/stress coupling between these two types of materials [2]. When a magnetic field is applied to ME composites, their ferromagnetic layers shrink or stretch due to the magnetostrictive effect; then, the resulting strain/stress is transferred to the piezoelectric material, leading to a voltage change [2]. Devices based on laminated ME structures have shown promising potential for use in magnetic sensors, acoustically driven antennas and power conversion devices due to their high energy conversion efficiency [1,2,3,4,6,7,8,9,10,11].

As a key component of ME structures, Fe-based amorphous alloys should possess a relatively high conversion efficiency between magnetic power and mechanical power. To develop such materials, the requirement is to balance two or more parameters, as relevant parameters in materials usually impact one another to a significant degree [1]. However, keeping a parameter unchanged while improving other correlated parameters is normally quite a challenging task, even more so to balance two main parameters in some energy-converting materials. Specifically, the magnetomechanical power conversion efficiency (*η*), defined by the ratio between the converted power and the input power and that can be expressed as $\eta =\frac{1}{1\;+\;1/(k^{2}Q)}$ [4,12], in magnetostrictive materials is based on two mutually exclusive, but related, parameters—the magnetomechanical coupling coefficient (*k*) and damping factor (*Q^−^*^1^) [2,4]. The *k* factor quantifies the ratio of the magnetic energy that is converted into mechanical energy or vice versa over a period in magnetostrictive devices [13,14,15]. The *Q* factor, a reciprocal of the damping factor, is another important parameter that quantifies the ratio between the power storage and the power loss [12,14]. The two factors can be used to characterize the magnetomechanical properties in Fe-based amorphous alloys [4]. The *k* and *Q* factors accompany each other in most magnetomechanical transduction materials. To increase *η*, *k*^2^*Q* needs to be maximized, although efforts to increase *k* often lead to a reduction in *Q* and vice versa. For example, the traditional annealing procedure usually causes a similar trend in changes to the *k*^2^ and *Q^−^*^1^ factors; this implies a small *η* value [1,2]. To enhance the efficiency a step forward, and to minimize the inevitable correlation between the two factors, further clarity is needed on the correlation in variations between *k* and *Q^−^*^1^.

Over recent decades, the influence of annealing on magnetic properties of Fe-based amorphous ribbons has been vastly studied [4,16,17,18]. It has been found that the annealing procedure can adjust the saturation magnetic flux density (*B_s_*), coercivity (*H_c_*), magnetic permeability (*μ_r_*) and the *k*, *Q* and *η* factors, but variations to these parameters are rarely independent [1,4,8,11,12,13,19,20,21,22,23,24,25,26,27,28,29,30,31,32,33,34,35]. Thus, an opportunity has arisen for researchers to explore ways to cooperatively elevate two or more parameters while avoiding negatively impacting others [1,2,3].

In this study, the influence of heat treatment on the magnetomechanical properties for FeSiB amorphous ribbons is investigated. The correlation between the *k* and *Q* (or *Q^−^*^1^) factors of such ribbons annealed at temperatures far below the crystallization temperature is discussed. The magnetic and magnetomechanical properties of single-foil ribbons and epoxy–ribbon laminates are also measured and compared in this work.

## 2. Experimental Method

**Figure 1:**
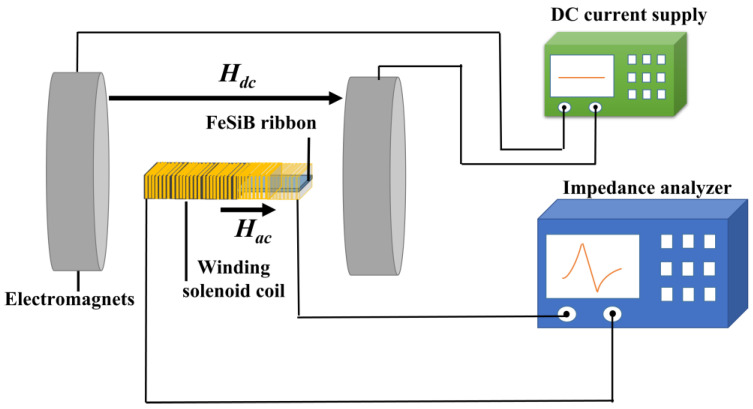
Schematic diagram of the experiment.

The samples used in this work to perform the experiments were Fe-based amorphous ribbons with a nominal composition of Fe_80_Si_9_B_11_ (at%). The length, width and thickness of a single ribbon were 40 mm, 5 mm and ~25 μm, respectively. The ribbons were annealed at different temperatures for 20 min in air using a muffle furnace. The annealed ribbons were inserted into a rectangular winding coil with an approximate size of 40 × 10 × 10 mm^3^ and were tightly wound into six layers using 32-AWG magnetic wires in copper. When amorphous ribbons are driven by a magnetic excitation near a mechanical resonant frequency, their impedance (and inductive component) is extremely sensitive to the applied DC field [5,7,36,37]. The inductance and the impedance of the coils with inserted ribbons were measured with a high-precision impedance analyzer (HP 4294A). The resonant frequencies (***f*_r_** at maximum impedance) and antiresonant frequencies (***f*_a_** at minimum impedance) on the impedance spectrum were also measured with the impedance analyzer under a DC magnetic bias field (*H_dc_*) along the longitudinal direction of the ribbons [6,38]. It is worth mentioning that the antiresonance occurred at a frequency above the actual mechanical resonance frequency when the impedance contribution from the ribbon minimized the total impedance [12]. The values of *k* were determined using resonant and antiresonant frequencies, while *Q* could be directly measured using the two poles (***f*_1_** at maximum inductance, ***f*_2_** at minimum inductance) on the inductance spectrum [4,12,38]. From the frequencies at *f_r_*, *f_a_*, *f*_1_ and *f*_2_, the values of *k* and *Q* were calculated using the following dependences: $k=\sqrt{\left(\pi^{2}/8\right)\left(1-f_{r}^{2}/f_{a}^{2}\right)}$ and $Q=f_{r}/\left(f_{2}-f_{1}\right)$, where $\Delta f=f_{2}-f_{1}$ also corresponds to the −3 dB bandwidth near the resonance [12]. A schematic diagram of the experiment is shown in Figure 1.

The magnetic permeability *μ_r_* of the Fe-based amorphous ribbon was an order of 10^4^–10^5^ [39]. When cut into the rectangular shape, the apparent magnetic permeability (*μ_app_*) along the long axis of the ribbon was several hundred due to the demagnetization field [40]. Furthermore, because the inductance of the winding coil was almost constant for a fixed frequency, this caused the measured inductance (*L*) values of the winding coil with an inserted Fe-based ribbon to increase with the increase in *μ_app_*, i.e., $L\propto \mu_{app}$.The values of Young’s modulus (*E*) for the ribbons with lengths matching the coils’ should have been approximately proportional to the square of *the* antiresonant frequency *f_a_* or the square of the resonant frequency *f_r_* [12,14,34], i.e., $E\propto f_{a}^{2}(or\;f_{r}^{2})$. The magnetic hysteresis loops of the ribbons annealed at different temperatures were measured using the vibrational sample measurement system (VSM, MicroSense EZ-7). The samples with dimensions of approximately 5 × 5 × 0.025 mm^3^ were prepared together in a furnace with the 40 mm long ribbons for the VSM measurements.

## 3. Theoretical Analysis and Measurement

### 3.1. Eddy Current Loss

The equivalent input loss factor (*ξ*) is defined as the inverse of the product of *k*^2^ and *Q*. It quantifies the ratio between the power conversion and the power loss in the magnetic-to-mechanical energy conversion. Following previous investigations [4,12,32,34,41], *ξ* could be written as

(1) $$\xi =\frac{1}{k^{2}Q}=c_{e}\mu_{0}\chi f_{r}+\frac{1}{k^{2}Q_{0}},$$

where *c_e_* is the loss coefficient for the eddy current in an individual ribbon, *χ* is the magnetic susceptibility of the ribbons and *Q*_0_ is the quality factor under magnetostriction-free conditions. From the right-hand side of Equation (1), the first term *c_e_* represents the energy loss for the dynamic magnetization procedure, which was affected by the magnetic properties of the ribbon; the second part represents loss not related to the eddy current. In single ribbons, the eddy current loss dominated after the heat treatment under an annealing temperature far below the crystallization temperature. Following Herzer et al. [42], the formula for *c_e_* was expressed as

(2) $$c_{e}=\frac{{\left(\pi t\right)}^{2}}{6\rho_{el}}\left(1+\frac{w^{2}}{{\left(w\cos\beta +t\right)}^{2}}\frac{m^{2}}{1-m^{2}}\right),$$

where *t* is the ribbon thickness, $\rho_{el}$ is the electric resistivity and *β* is the angle between the average magnetic anisotropy and the ribbon direction. $m=J_{H}/J_{s}$ is the average longitudinal magnetization (*J_H_*) normalized to the saturation magnetization (*J_S_*). *w* is the magnetic domain width; the formula was given as [42]

(3) $$w^{2}=\frac{8L_{ex}t}{N_{zz}\sin^{2}\beta +N_{yy}\cos^{2}\beta },$$

where $L_{ex}=\sqrt{A/K}$ is the magnetic exchange length. *A* is defined as the exchange stiffness and *K* is the anisotropy constant. *N_zz_* and *N_yy_* are the demagnetization factors along the longitudinal and width directions of the ribbon, respectively. With the help of Equations (2) and (3), the contribution of the eddy current in Equation (1) could be rewritten as

(4) $$\xi_{eddy}=\frac{{\left(\pi t\right)}^{2}}{6\rho_{el}}\mu_{0}\chi f_{r}+\frac{{\left(\pi t\right)}^{2}}{6\rho_{el}}\frac{m^{2}}{1-m^{2}}\frac{w^{2}}{{\left(w\cos\beta +t\right)}^{2}}\mu_{0}\chi f_{r}.$$

Taking into account that the average anisotropy angle *β* was close to zero for ribbons annealed at low temperatures, Equation (4) was rewritten as

(5) $$\xi_{eddy}=\frac{{\left(\pi t\right)}^{2}}{6\rho_{el}}\mu_{0}\chi f_{r}+\frac{\pi^{2}}{6\rho_{el}}\frac{m^{2}}{1-m^{2}}{\left(\frac{1}{t}+\frac{1}{w}\right)}^{-2}\mu_{0}\chi f_{r}.$$

Since the magnetic domain width was usually much larger than the ribbon thickness in low-temperature annealed samples, the assumption *w* >> *t* could be considered in the calculation. Thus, Equation (5) could be rewritten as

(6) $$\xi_{eddy}=\frac{1}{k^{2}Q}=\frac{{\left(\pi t\right)}^{2}}{6\rho_{el}}\left(1+\frac{m^{2}}{1-m^{2}}\right)\mu_{0}\chi f_{r}.$$

The equivalent loss factor induced by the eddy current loss for the ribbons annealed at low temperature was dominated by the variation in *χ* and *f_r_* for the ribbons.

### 3.2. Magnetic and Magnetomechanical Properties

**Figure 2:**
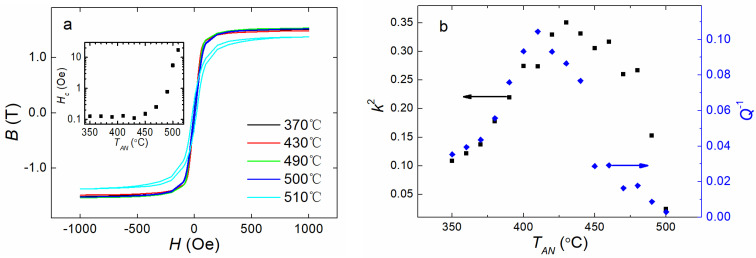
(**a**) The hysteresis loops of FeSiB amorphous ribbons obtained after annealed at different temperatures, from 370 °C to 510 °C, for 20 min in air; the inset figure is the annealing temperature (*T_AN_*) dependence of coercivity *H_c_*. (**b**) Damping factors (*Q^−^*^1^) and the square of magnetomechanical coupling (*k*) as a function of *T_AN_* for 20 min in air. *k* was acquired at its maximal values with external DC magnetic bias field *H_dc_*. The values of *Q^−^*^1^ were measured under the same *H_dc_* when *k* was maximized.

It was assumed that the hysteresis loops of the magnetic materials could provide important information on the magnetic properties of the materials. Figure 2a shows the DC magnetic hysteresis loops of the samples annealed in air at different annealing temperatures (*T_AN_*) for 20 min. The loops showed small values of remnant magnetization and *H_c_* for all the samples, suggesting that the samples retained good soft magnetic properties after the heat treatment in air. As shown in the inset in Figure 2a, the values of *H_c_* mostly stayed close to 0.1 Oe for *T_AN_* = 370 °C to 450 °C. However, *H_c_* began to grow sharply when *T_AN_* exceeded 450 °C. In this case, the change in the chemical concentration causing the ordered clusters and the subsequent topological and chemical long-range orderings on the surfaces of the ribbons resulted in variations in the values of *H_c_* [28,30,31,33,41,43]. When *T_AN_* was above 500 °C, *H_c_* showed a high increase, which suggested further deterioration in the soft magnetic properties. This was due to (1) an increase in the fraction of the surface crystallization at high *T_AN_* and (2) the film of boron oxides that formed due to the excessive presence of boron atoms that were separated from the α-Fe crystallites, since the α-Fe crystallites had a much lower solubility effect on B than that of amorphous Fe [4,22,27,28,30,33]. Consequently, the magnetic domains in the amorphous remainders were thinned and turned toward the out-of-plane direction through compression stress induced by the surface crystallization and surface oxidation films [4,22,27,28,30,33], leading to an increasing in *H_c_*.

In our previous work [4], we reported on the *T_AN_* dependency of *k* and *Q* for single FeSiB ribbons; the value of *k* reached its maximum at approximately a *T_AN_* of 430 °C, while *Q* showed a minimal value at approximately a *T_AN_* of 410 °C. The *k*^2^ and *Q^−^*^1^ evolutions after annealing at various temperatures *T_AN_* are shown in Figure 2b. In the low *T_AN_* region, below 400 °C, with increasing *T_AN_*, both *k*^2^ and *Q^−^*^1^ increased at almost the same pace, indicating that *k*^2^ and *Q^−^*^1^ were correlated in the low annealing temperature region. For higher *T_AN_*, from 400 °C to 500 °C, the variation in *k*^2^ and *Q^−^*^1^ diverged, suggesting that the correlation level of the two parameters dropped significantly. Furthermore, as a result of the collective changes in *k*^2^ and *Q^−^*^1^ for low *T_AN_*, below 400 °C, the equivalent input loss *ξ* should have stayed invariable for low *T_AN_*, below 400 °C, as reported by our earlier investigations [4].

### 3.3. Softening of Magnetic and Elastic Properties

**Figure 3:**
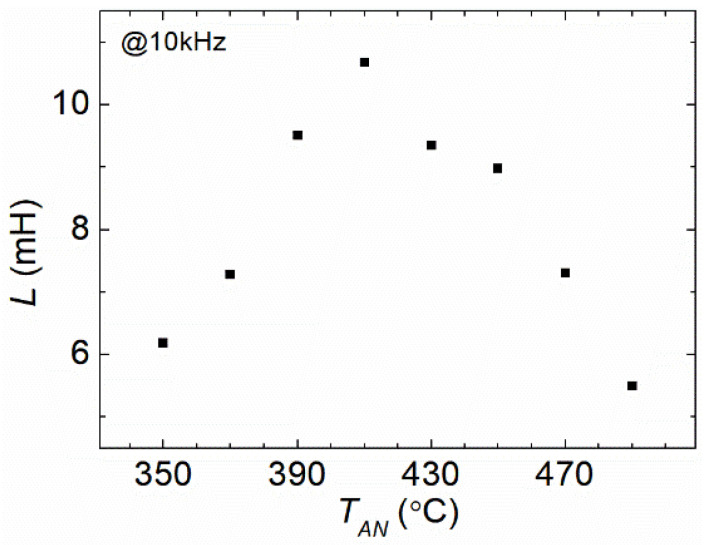
Variations in inductance (*L*) of the winding coil with an inserted FeSiB ribbon annealed for 20 min at different annealing temperatures *T_AN_*, from 350 °C to 490 °C.

Figure 3 shows the measured values of the inductance (*L*) at 10 kHz after isothermal annealing at various temperatures *T_AN_*. It could be observed that *L* increased with *T_AN_* in the region from 350 °C to 410 °C and then decreased for *T_AN_* from 420 °C to 490 °C. As mentioned above in the Section 2, the variation in *L* values suggested a collective change in the apparent magnetic permeability (*μ_app_*) of the ribbon [44], i.e., $L\propto \mu_{app}$.

**Figure 4:**
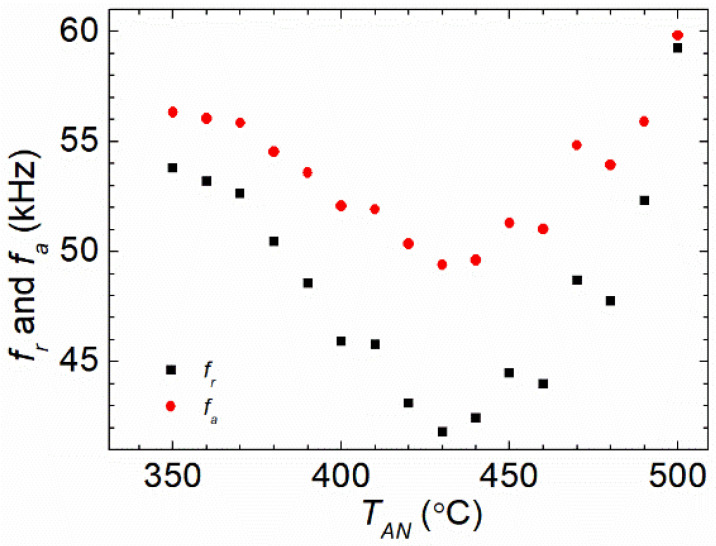
The variation in the resonant frequencies (*f_r_*) and antiresonant frequencies (*f_a_*) of the winding coil with an inserted FeSiB ribbon annealed for 20 min at different annealing temperatures *T_AN_*, from 350 °C to 500 °C. The *f_r_* and *f_a_* on the motion impedance curve were measured with the impedance analyzer under a DC magnetic bias field (*H_dc_*) along the longitudinal direction of the ribbons.

Figure 4 shows the *T_AN_* dependence of resonant *f_r_* and antiresonant *f_a_*. It could be stated that in the low *T_AN_* region, both *f_r_* and *f_a_* decreased with the increase in *T_AN_*, reaching a minimal value at 410 °C. Then, they rose as *T_AN_* continued to increase. The values of Young’s modulus (*E*) in the ribbons with lengths matching those of the coils should have been approximately proportional to the square of *the* antiresonant frequency *f_a_* or the square of the resonant frequency *f_r_* [12,14,34], i.e., $E\propto f_{a}^{2}(or\;f_{r}^{2})$. By comparing the behavior of *L* and *f_a_* (*f_r_*) versus *T_AN_*, it could be seen that the two parameters exhibited a respective extreme value as the function of *T_AN_*. However, the curves of *L* and *f_a_* (*f_r_*) showed a lag of 20 °C versus *T_AN_* to reach their maximum/minimum values.

By comparing the *T_AN_* dependence of *L* with the *T_AN_* dependence of the *Q^−^*^1^ factors, it could be observed that the variations in *L* and *Q^−^*^1^ with the increase in *T_AN_* were almost the same below 400 °C; *f_a_* (*f_r_*) had a similar profile to the *k* factor. According to Equation (6), the trends of *k* and *Q* were recognized as the competition between the magnetic and mechanical properties, *χ* and *f_a_* (*f_r_*). Since the *L* curve and *Q^−^*^1^ curve shared the same trend and the same extreme value point of *T_AN_* below 410 °C while the *f_a_* (*f_r_*) curve and the *k* curve showed an inverse trend and the same extreme value point of *T_AN_* below 430 °C, it was probable that *L* or *μ_app_* were dominant in the values of the *Q^−^*^1^ factors, while *f_a_* (*f_r_*) or *E* had a dominative effect on the *k* factor for the low *T_AN_* below 410 °C (for *L* and *Q*) or 430 °C (for *f_a_* and *k*). Moreover, because *k* and *E* were related to each other, the annealing experience through *α* relaxation had a significant influence on the softening of elasticity below 430 °C [4]. For higher *T_AN_*, because of the emergence of the surface crystallization and the long-range chemical orderings with B oxidation during the annealing procedure, the competition of magnetism and elasticity became much more complex, leading to the divergence between the *k*^2^ curve and *Q* curves, as well as between the *f_a_* (*f_r_*) curve and the *L* curve versus *T_AN_* [4,28,30,31,33,35,41].

Below 410 °C-*T_AN_*, the *β* relaxation in the Fe-deficient zones was dominant and led to the chemical short-range ordering (CSRO) in Fe-rich zones with doped B atoms through diffusion [4]. In addition, the topological short-range ordering (TSRO) also occurred at this low *T_AN_* region as another result of *β* relaxation, leading to the release of internal stress, both of which weakened the pinning effect of the magnetic domain through defects in the sample [4]. The expanding speed of the magnetic domain increased, therefore, i.e., *L* (*μ_app_*) increased. For *T_AN_*, from 450 °C to 500 °C, the sharp decrease in *L* (*μ_app_*) may have occurred due to the emergence of surface crystallization together with more severe surface oxidation (such as Fe-O, B-O, silica) that led to the appearance of the grain boundary, as well as the magnetic anisotropy deviating from the long axis direction, both of which inhibited the movement or rotation of the magnetic moment of the magnetic domain, resulting in the smaller variation rate of the magnetic moment. The increase in *H_c_* above 450 °C also coincided with the decrease in *L* (*μ_app_*), suggesting the deterioration of the soft magnetic properties in the high *T_AN_* region.

The decrease in *E* in the low *T_AN_* region suggested that the deformation quantity of the ribbon decreased with the increasing *T_AN_*. According to previous studies [4,35], as a result of *β* relaxation for low *T_AN_*, besides the increase in the Fe–Fe bond caused by the CSRO, the TSRO could also be triggered through *β* relaxation close to the cluster–matrix boundaries. The TSRO initiated a decrease in the ductility of the sample; thus, the sample became “more flexible” when stretched. Consequently, *E* (*f_a_* and *f_r_*) decreased after annealing at a relatively low temperature, and reached its minimum at 430 °C, as shown in Figure 4. The *E* (*f_a_* and *f_r_*) vs. *T_AN_* curve switched to an increasing trend when *T_AN_* exceeded 430 °C. This could be attributed to the apparency of *α* relaxation, which was more intense and usually occurred at a high *T_AN_*. This *α* relaxation further enhanced the diffusion of the atoms, affecting the atomic orderings in a larger scale and usually reshaping the clusters. Fe or metalloid atoms experiencing long-term relaxation in the B-rich area led to the generation of some clusters with high elasticity, which increased the values of *E*. In addition, because the annealing procedure in this article was performed in air, the oxidation was stronger and penetrated deeper into the ribbons at a high *T_AN_*; this also had a strong influence on the rigidity of the ribbon [4,20].

From Figure 3 and Figure 4, it could be obtained that the variation trends in magnetic susceptibility *χ* (*χ* was also approximately proportional to *L* in Figure 3) and resonant (antiresonant) frequency *f_r_* (*f_a_*) were quite similar, but opposite for low *T_AN_* from 350 °C to 400 °C; the product of *χ* and *f_r_* (*f_a_*) remained close to the constant. Moreover, the measured *η*, relating to *k*^2^*Q*, was nearly a constant value in this region of *T_AN_*, following our previous investigations [4,8]. Therefore, according to Equation (1), it could be inferred that the loss factor *c_e_* that represented the eddy current loss should have been mostly unchanged for low *T_AN_* from 350 °C to 400 °C for the single FeSiB ribbons. This was consistent with our analysis in the theoretical section as well.

When *T_AN_* approached the crystallization temperature (*T_x_*) of the FeSiB glassy metals, the size of the magnetic domain decreased due to the change in surface stress [4,31,33]. According to Herzer et al. [4,12,32,34,41,43], the loss factor *c_e_* is related to the width of the magnetic domain *w*, following Equation (2). Therefore, the collectively changing behavior of *k*^2^ and *Q^−^*^1^ suggested that the improvement in the soft magnetic properties may have derived from the increase in the number of activated magnetic units rather than variations in the width of the magnetic domains in the low *T_AN_* region. Thus, the values of *ξ* for the ribbons annealed at low *T_AN_* remaining constant had more to do with the increase in the quantity of magnetic units rather than the change in the domain size.

### 3.4. Time–Temperature Equivalence for Annealing

**Figure 5:**
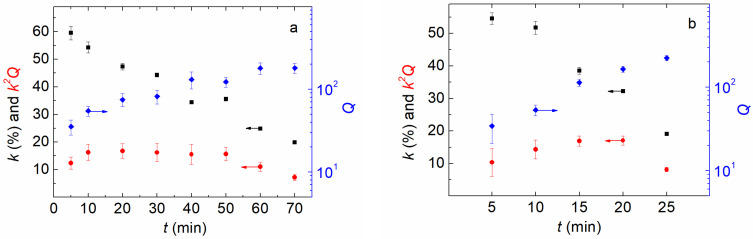
The magnetomechanical coupling coefficient (*k*) for FeSiB ribbons as a function of the annealing time (*t_AN_*) at annealing temperature of (**a**) 470 °C and (**b**) 490 °C. The blue and red curves represent the values of the quality factors (*Q*) and efficiency factors (*k*^2^*Q*) associated with the maximum values of coupling coefficient *k*, respectively.

Figure 5a,b display the variations in the *k*, *k*^2^*Q* and *Q* factors for different annealing times *t_AN_* at *T_AN_* ranging from 470 °C to 490 °C, respectively. A significant decrease in the *k* factor with the increase in the *Q* factor occurred with the increase in *t_AN_* for a *T_AN_* of 470 °C and 490 °C. The curve for *k* and *Q* showed a cross profile at *t_AN_* = 40 min and 15 min, respectively. After the ribbons were annealed for 40 min at *T_AN_* = 470 °C and 15 min at *T_AN_* = 490 °C, *k* decreased from approximately 60% to 40%, while the values for *Q* increased close to 200. The equivalent input loss factors *ξ* showed a maximum value when the *t_AN_* was 20 min for both samples, which suggested the optimal annealing time (20 min) for annealing at 470 °C and 490 °C. Based on the data mentioned above, it was implied that the decrease in *k* was caused by the surface oxidation and surface crystallization that induced the increase in coercivity through the out-of-plane magnetic anisotropy. The increase in *Q* factors might have been due to the long-range orderings that took place on the surface regions of the ribbons.

The *Q* factor represents the quality of mechanical performance in our FeSiB ribbons; a high value in the *Q* factor indicates a high ratio between the storage power and power loss. Figure 5a,b show that the overall trend of the *k*, *k*^2^*Q* and *Q* curves at 470 °C *T_AN_* was similar to that at 490 °C *T_AN_*, separately. Moreover, the increase in *T_AN_* from 470 °C to 490 °C narrowed the *T_AN_* window before the magnetomechanical performance visibly deteriorated. In terms of the *η* factors, it was similar to the increase in *T_AN_* for a constant *t_AN_* or to increase in *t_AN_* at a fixed *T_AN_*, which suggested that *T_AN_* and *t_AN_* had an equal effect on the *η* factor in the heat treatment procedure for the FeSiB ribbons to some extent. However, the equivalence of *t_AN_* and *T_AN_* in terms of the magnetomechanical power conversion efficiency seemed to exist only at relatively higher *T_AN_*; we did not observe this equivalent with *T_AN_* below 400 °C.

### 3.5. Magnetomechanical Properties in Epoxy–Ribbon Composites

**Figure 6:**
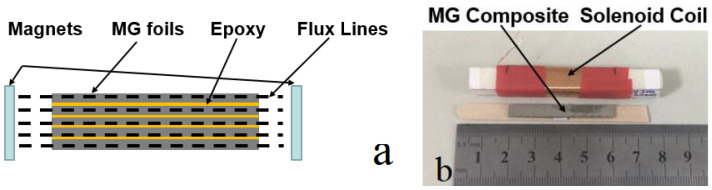
(**a**) Schematic diagram of a laminated composite consisting of magnetostrictive amorphous FeSiB ribbons bonded with epoxy resin. (**b**) Photo of laminated metallic glass (FeSiB) ribbon composites and the winding coil used to generate the AC magnetic field.

**Figure 7:**
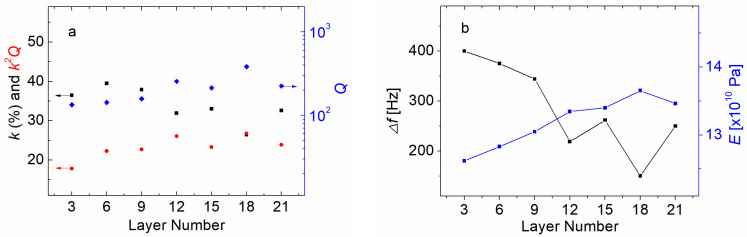
(**a**) The black curve is the magnetomechanical coupling factor (*k*) for laminated metallic glass composites consisting of magnetostrictive amorphous FeSiB ribbons as a function of the FeSiB layer number. The blue and red curves represent the values of the quality factors (*Q*) and efficiency coefficients (*k*^2^*Q*) associated with the maximum values of coupling coefficient *k*, respectively. (**b**) The trend of the −3 dB bandwidth (∆*f*) and Young’s modulus (*E*) for laminated FeSiB ribbon composites as a function of the FeSiB layer number, where $\Delta f=f_{2}-f_{1}$ (*f*_1_ at maximum inductance, *f*_2_ at minimum inductance).

A schematic diagram of a laminated composite consisting of magnetostrictive amorphous FeSiB ribbons bonded with epoxy resin is given in Figure 6a. Several FeSiB foils were fabricated using hot-pressing techniques and an A–B part epoxy. The ratio between the epoxy (A part) and the curing agent (B part) should be 3:1, but in this case the curing part is a little bit less than the should-be value. Figure 6b is a photo of the laminated FeSiB composites; we applied a DC magnetic field along the laminated composite, as shown in the upper part of Figure 6b. The ratio between the quantity of the magnetostrictive layers and the quantity of epoxy resin varied during the investigation of the changes in *k*, *Q* and *k*^2^*Q* in the FeSiB laminated composites with different foil numbers. The results are given in Figure 7a. It was found that as the number of FeSiB layers increased, the *k* factors increased slightly and then decreased rapidly, while the *Q* factors showed an overall increasing trend to a maximum close to 400, corresponding to a foil number from 18 to 21 in the FeSiB laminates. The efficiency factor *k*^2^*Q* first increased in the presence of more FeSiB ribbon layers and then reached a relatively stable value at approximately 25, corresponding to an FeSiB foil number from 12 to 21. Figure 7b shows the trend of the −3 dB bandwidth (Δ*f*) and *E* for the FeSiB laminated composites with different foil numbers.

In Figure 7a, it was observed that when the foil number was small, there was no obvious similarity or correlation between the *k* and *Q* factors as the FeSiB layer number varied. The *k* and *Q* in the laminated composites with nine layers, six layers and three layers of FeSiB ribbons were examples. However, for the laminated composites with more foil number of 12 layers, 15 layers, 18 layers and 21 layers, the data suggest that there was a mutually exclusive relationship between the *k* and *Q* factors. This caused the *k*^2^*Q* factors to reach a relatively stable state, while the *k*^2^*Q* factors did not continue to increase with the increase in FeSiB layers. For the record, we needed to particularly emphasize that the ratio between the epoxy and curing agents was *not* 3:1. The amount of curing agent was reduced, giving a ratio of more than 3:1 between the epoxy and the curing agent, so the epoxy resin was not fully solidified for the laminated samples. Thus, the mechanical loss due to the interfriction increased compared to the samples with fully cured epoxy. Unlike the eddy current loss in the single-foil ribbons, the dominant loss in the FeSiB laminates was ascribed to the mechanical loss that triggered the temperature rise when the laminated composites were driven under high-power conditions.

According to previous research [4,31], as *T_AN_* approached *T_x_*, the size of the magnetic domain decreased due to the change in surface stress. As mentioned above, the efficiency of a single-foil ribbon experienced a low *T_AN_* that remained constant, and this was more due to the increase in the number of magnetic units rather than the change in the magnetic domain size. In contrast, the increase in the *k*^2^*Q* factors at *T_AN_* above 450 °C was due to the reduction in magnetic domain size. Following Equation (1), because the *k*^2^*Q* factors of the laminated FeSiB composites were approximately constant for a high layer number of laminates, and because the variation trend of *E* (associated with *f_r_* and *f_a_*) and the bandwidth (associated with χ) of the laminated FeSiB composites remained consistent with each other for all the foil numbers, the *c_e_* of the laminated composites should have also remained unchanged. As the foil number of the FeSiB laminates decreased, the volume proportion of magnetostrictive materials (FeSiB) in the laminated composite gradually increased. Using a similar analysis principle to Figure 7a, the reason for the constant values in the *k*^2^*Q* factors for the ribbons with foil numbers ranging from 12 to 21 was probably due to the increase in the number of ribbons rather than the change in the relative fraction of the ribbons. The reason for the change in the *k*^2^*Q* factors for the laminates with foil numbers ranging from 3 to 12 was due to the variation in the relative volume fraction of FeSiB ribbons rather than a variation in the ribbon number. This indicated that the change in the *k*^2^*Q* factors between the laminates with ribbon numbers ranging from 3 to 12 derived from the relative size of the magnetic units, which probably corresponded to the relative volume fraction of the FeSiB ribbons in the laminates, while the constant *k*^2^*Q* factors between the 12-layer and 21-layer laminates might have arisen from the change in the number of magnetic units rather than the change in the relative volume of magnetic units [45].

## 4. Conclusions

In summary, we investigated the influence of magnetic and elastic properties on magnetomechanical coupling factors (*k*) and damping factors (*Q^−^*^1^) by measuring the resonant and antiresonant frequencies from the motion impedance and inductance spectrum of a winding coil with an annealed FeSiB ribbon. We could draw the following conclusions:

1. Through annealing under different temperatures, it was found that the dynamic magnetic and elastic properties in the Fe_80_Si_9_B_11_ ribbons varied correlatively with the annealing temperature. However, the resonant frequency reached its minimum value at 430 °C, with a lag of 20 °C, when the magnetic parameter reached its minimum at 410 °C, coinciding with the behaviors of *k* and *Q^−^*^1^ when the annealing temperature changed.

2. It was found that the annealing temperature and annealing time equally impacted the heat treatment procedure for the FeSiB ribbons in maximizing the magnetomechanical power efficiency. However, the equivalence was correct only when the annealing temperature was high enough for the long-range orderings to occur on the surface region of the ribbons.

3. For the FeSiB laminated composites, when the foil number ranged from 12 to 21, the trends of *k* and *Q* were synchronously related but opposite. This suggested that the behavior of the *k* and *Q* factors in the laminates with foil numbers ranging from 12 to 21 was at some point in analogy with the behaviors of the *k* and *Q^−^*^1^ factors in the single-foil FeSiB ribbons with various annealing temperatures ranging from 350 °C to 400 °C.

## Data Availability

Not applicable.
